# Determination of particle size and number concentrations in gold nanoparticle mixtures: an inter-method study using four analytical techniques (DLS, PTA, SEM and spICP-MS)

**DOI:** 10.1039/d6na00161k

**Published:** 2026-07-31

**Authors:** Birgit Hetzer, Alexandra Müller, Ann-Katrin Meinhardt, Volker Gräf, Elke Walz

**Affiliations:** a Max Rubner-Institut, Federal Research Institute of Nutrition and Food, Department of Food Technology and Bioprocess Engineering Haid-und-Neu-Str. 9 76131 Karlsruhe Germany birgit.hetzer@mri.bund.de

## Abstract

The present study was conducted to determine the size of gold nanoparticles and their respective number concentrations in mono-, bi- and trimodal suspensions using dynamic light scattering (DLS), particle tracking analysis (PTA), scanning electron microscopy (SEM) and single particle inductively coupled plasma mass spectrometry (spICP-MS). This intercomparison approach aimed to test the various analysis methods for their suitability to characterise as well as to distinguish the different nanoparticle fractions in the samples. Thereby, the advantages and limitations of both the employed measurement techniques and the colloidal suspensions containing different concentrations of AuNPs became apparent. Here, DLS and PTA provided reasonably good data on the mean hydrodynamic diameter. In addition, PTA was able to determine the total amount of nanoparticles in all monomodal and in six out of eight multimodal suspensions. However, both methods failed to quantify the number of particles for each individual fraction. Using SEM, the mean sizes of the monomodal suspensions and the sizes of all individual fractions in the multimodal samples could be determined without difficulty. Furthermore, relative particle number ratios of the individual fractions could be obtained, even in the absence of data on the total particle concentration. Finally, despite having its own challenges regarding calibration, spICP-MS was the only method investigated capable of determining not only the mean diameter but also the particle concentration and simultaneously distinguishing and determining the individual fractions in all multimodal samples. This study demonstrated that experimental scenarios exist where individual methods reach their limits even by using well-defined gold nanoparticles of known sizes and concentrations. Therefore, various complementary methods are necessary for nanoparticle analysis to obtain the most comprehensive picture.

## Abstract 2

Reliable fraction-resolved analysis of particle size and number concentration of multimodal gold nanoparticle mixtures (30–100 nm) requires complementary methods. In this inter-method study, spICP-MS provided the most comprehensive information.

## Graphical abstract

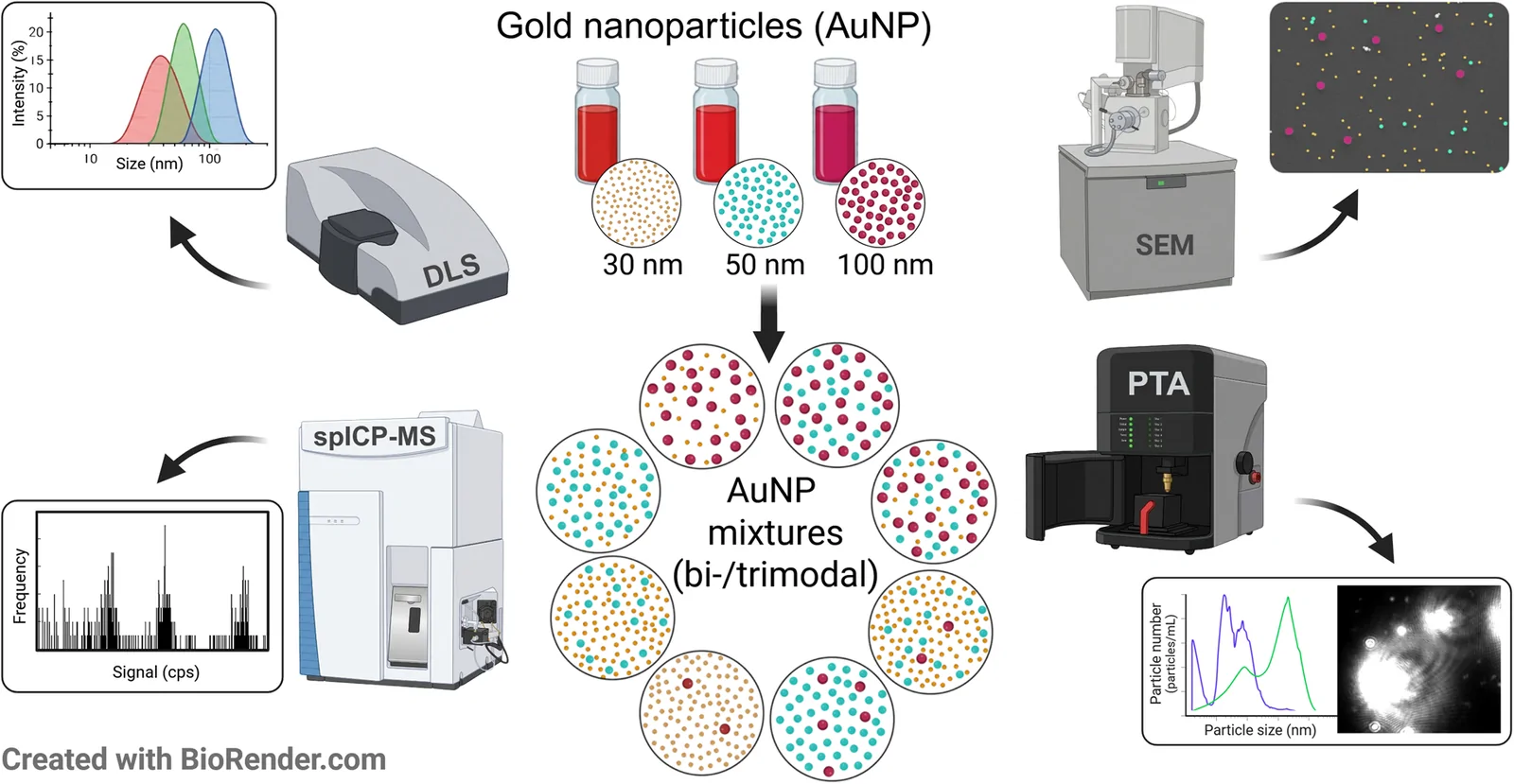

## 1 Introduction

Nanomaterials are used in a variety of sectors, including packaging, cosmetics, food, and dietary supplements.^1–7^ In the food sector, the legal situation requires that (intentionally manufactured) nanoparticles must be identified and quantified in order to fulfil labelling obligations with regard to the ‘Food Information to Consumers’ (FIC) regulation [1169/2011].^8^ If a food contains an engineered nanomaterial (ENM), the name of the ingredient concerned shall be followed by the term ‘(nano)’ in the list of ingredients. The term ‘engineered nanomaterial’ is specifically defined in Regulation (EU) 2015/2283, commonly known as the ‘Novel Food’ regulation.^9^ This regulation includes only intentionally manufactured materials and no materials of natural and process-derived origin. Currently, neither FIC nor ‘Novel’ Food regulation specifies a concentration threshold for the nanoparticle content above which labelling becomes mandatory. This is in contrast to the general legally non-binding EU's nano definition recommendation,^10^ which states that a material is a nanomaterial if 50% or more of the particles have one or more external dimensions in the size range 1 nm to 100 nm with regard to the number-based particle size distribution.

In order to comply with the labelling obligation, both manufacturers and control authorities must be able to prove whether nanomaterials are present according to the EU's nano definition recommendation. To date, legally mandated analytical techniques for particle size monitoring of (food-related) ENMs have been established so far only to one specific nanoparticulate material: iron hydroxide adipate tartrate (IHAT), the first ‘Novel Food’ nanomaterial to be launched on the EU market, characterised by dynamic light scattering (DLS) and transmission electron microscopy (TEM).^4,11^

For orientation, both the European Food Safety Authority (EFSA) and the Organisation for Economic Co-operation and Development (OECD) have published general guidelines, including recommendations on analytical methods.^12–14^ In order to adequately characterise the various ENMs' physico-chemical properties, such as chemical composition, particle size and concentration, shape and morphology and the level of aggregation or agglomeration, a multi-method approach with dedicated nanoanalytical techniques is needed to get ‘the full picture’.^1,15–18^ With regard to the available nanoparticle analysis portfolio, a distinction is often made between screening methods and confirmatory methods. Screening methods include techniques like dynamic light scattering (DLS), single particle ICP-MS (spICP-MS), particle tracking analysis (PTA, also known as nanoparticle tracking analysis (NTA)), centrifugal sedimentation (DCS) or surface area analysis (*e.g.* gas adsorption method based on the Brunauer–Emmett–Teller theory, BET). They are generally used to assess rapidly whether nanoparticles might be present. Furthermore, UV-Vis spectroscopy has been reported as a promising low-cost and rapid optical approach for the simultaneous estimation of nanoparticle size and concentration.^19,20^ Recent developments in machine learning and artificial intelligence-assisted spectral analysis may further enhance the capabilities of this technique by enabling a more advanced evaluation of optical spectra, including the estimation of particle size distributions and concentrations in plasmonic nanoparticle systems.^21–23^

Confirmatory methods include microscopy-based methods like atomic force microscopy (AFM) and variations of electron microscopy (EM) – often combined with techniques for composition analysis (*e.g.* energy dispersive X-ray spectroscopy, EDX). They provide a definitive identification of the nanoparticles (even in particle aggregates), thus supporting the findings of screening methods. The list of the above-mentioned methods is non-exhaustive – an overview of available methods can be found in the appendix of the EFSA guidance on nanomaterial risk assessment^12^ or the Joint Research Centre (JRC, the European Commission's science and knowledge service) guidance on the implementation of the nano definition.^24^ Depending on the objective and nanomaterial application, characterisation by a screening method can often be sufficient. Nevertheless, in compliance with regulatory requirements, a more reliable and valid analysis with confirmatory methods is essential.

Due to a lack of Europe-wide standardisation, fit-for-purpose strategies and detection methods for the *in situ* analysis of nanoparticles in complex matrices – not only restricted to food – are still in the process of development and adaptation. For accurate characterisation, nanoparticles must typically be isolated from the complex food matrix. This isolation process must preserve the original particle characteristics – such as size, size distribution, morphology, and the extent of agglomeration or aggregation – while preventing dissolution or other physicochemical alterations, which is extremely challenging due to the complex nature of food matrices. The JRC is significantly supporting the development of sample preparation and analysis methods, *e.g.* by initiating projects and interlaboratory comparisons (ILCs) within the scope of the ‘Nanomaterials in Food Laboratory Group’ (NIF-LAG). Important outcomes in this context are methods for the determination of the transport efficiency in spICP-MS, particle size analysis of food-grade titanium dioxide in confectionery products and IHAT in a food supplement.^4,25,26^

In addition, the ‘Nanotechnologies working group’ (Technical Committee 352) of the European Committee for Standardization (CEN) has developed a Technical Specification entitled ‘Guidelines for sample preparation, detection, identification and characterisation by spICP-MS and EM-EDX of nano-objects in inorganic additives incorporated in food matrices’.^27^ Within other frameworks and platforms (*e.g.* European Metrology Programme for Innovation and Research, EMPIR; Versailles Project on Advanced Materials and Standards, VAMAS) other projects are initiated in order to develop traceable measurement procedures for nanoparticle characterisation in general, including *e.g.* the project ‘nPSize – improved traceability chain of nanoparticle size measurements’, which focusses on EM, AFM, spICP-MS and small angle X-ray scattering (SAXS).^28^

Interlaboratory comparisons (ILCs) and other proficiency testing schemes offer valuable information for selecting appropriate analytical methods by highlighting the conditions under which a chosen method performs reliably and where it may fail to meet performance criteria. Over the last fifteen years, numerous studies have been conducted to analyse nanoparticles. These studies have applied a variety of methods individually or in combination, focussing on different analytical aspects: some studies focused on determining the particle size or particle size distribution of monomodal samples containing gold, silver, silica, titanium dioxide or other metal oxide nanoparticles or polymeric nanomaterials,^29–47^ whereas other studies aimed on the analysis of more complex samples containing these nanomaterials with bi- or multimodal size distributions.^18,35,48–59^ It has to be mentioned that the listed studies represent only a selection and are not exhaustive.

One of the remaining key challenges is the limited availability of certified nanoparticulate reference materials, especially with respect to particle number concentrations and the use of engineered nanomaterials in the food sector. Furthermore, only a handful of analysis techniques are applicable for nanoparticle quantification and available practical findings and acquired knowledge from existing studies become scarce. Exemplarily, a few studies about the quantification of monomodal nanoparticle samples, mainly of metallic origin, can be mentioned here.^60–66^ Additionally, there are some investigations about the analysis of bi- or multimodal samples, measuring the nanoparticle content as mass concentration,^67^ relative particle concentration^52^ or number concentration.^57,68^

Although the above-mentioned studies highlight the capabilities of the analytical approaches and cover a wide field of applied analytical techniques and nanomaterials, there is still a lack of more comprehensive method comparison studies that take into account both particle size and number concentration determination of multimodal samples.

In order to fill this gap, this study evaluates four different analytical techniques for characterising gold nanoparticle (AuNP) suspensions. In a four-step design monomodal samples of each size class and eight different multimodal mixtures of 30, 50, and 100 nm AuNPs were analysed. Four methods were applied: Dynamic Light Scattering (DLS), Particle Tracking Analysis (PTA), single particle ICP-MS (spICP-MS), and Scanning Electron Microscopy (SEM) and tested for their ability to distinguish nanoparticle populations in complex samples. The goal was to assess each method's effectiveness in differentiating size distributions and to provide each fraction's particle number concentration (PNC) in multimodal suspensions. The results were evaluated with regard to strengths and limitations of each technique and practical considerations, offering insights for nanoparticle research and quality control. In this study, all measurements were based on the same aliquot of a single production batch and the mixtures prepared from it. Although method-specific dilutions were required for the individual analytical techniques, these were all prepared from the same starting material, thereby minimising variability associated with preparing, shipping and handling separate source materials.

## 2 Materials and methods

### 2.1 Samples

Gold nanoparticle suspensions (‘nanospheres’, polymer-coated, PEG Carboxyl, Ultra Uniform, ‘AUXU’/‘AUXS’, nanoComposix Inc., San Diego, California, United States) with three different particle sizes (30 nm, 50 nm and 100 nm) were used for the experiments.^69^ The monomodal samples (AUXU30, AUXU50, AUXS50 and AUXU100) (Table 1) were treated for 30 s at 80 W and 45 kHz in an ultrasonic bath (USC300THD, VWR, Darmstadt, Germany) and vortexed for 30 s.

**Table 1:** Summarised information and certified parameters for the monomodal AuNP suspensions given in the manufacturer's Certificate of Analysis (CoA)

	AUXU30	AUXU50	AUXS50	AUXU100
LOT number	JSF0123	SZD0103	JLC0238	PSK0236
Mean diameter (TEM)^a^ (nm)	28.2 ± 0.6	49.8 ± 1.8	51.0 ± 1.9	102.2 ± 4.2
Hydrodynamic diameter^b^ (nm)	36	59	n. a.	107
Mass concentration^c^ (ppm)	54	52	0.1342	53
Particle number concentration^d^ (particles per mL)	2.4 × 10^11^	4.2 × 10^10^	1.1 × 10^8^	4.9 × 10^9^

a Based on the measurement of 100 individual particles imaged with TEM (JEOL 1010).

b Measured with DLS (Malvern Zetasizer Nano ZS).

c Measured with ICP-MS (Thermo Fisher X Series 2).

d For AUXU30/50/100: calculated value based on the mass concentration determined by ICP-MS; for AUXS50: measured with spICP-MS (Agilent 7800).

For SEM analysis all samples were prepared directly from the original AuNP stock suspensions without further dilution. For DLS, PTA, and spICP-MS, the three AUXU samples were diluted 1 : 50 (w/w) with 2 mM sodium citrate buffer; the number standard AUXS was measured undiluted in PTA and DLS due to its relatively low concentration. The 2 mM sodium citrate solution was filtered through a 20 nm syringe filter (Anotop™ 25, Whatman™, Dassel, Germany) prior to use. For spICP-MS analyses a second dilution step was required (see Section 2.5).

Further information about the method-specific handling of the samples is provided in the following methods sections of DLS, PTA, spICP-MS and SEM analysis individually (2.3–2.6).

### 2.2 Experimental setup

Measurements with the four chosen techniques were carried out in four steps: in step 1, the monomodal particle number standard suspension (AUXS50) was tested using PTA and spICP-MS in order to verify the certified number concentration. In step 2, the three monodisperse AuNP samples (AUXU30, AUXU50 and AUXU100) were measured individually with each technique in order to characterise the source material. In step 3, bi- and trimodal particle mixtures with equal particle number concentrations of each particle fraction included were prepared from the three “AUXU” stock suspensions and analysed (mixes 1–4). In step 4, bi- and trimodal particle mixtures with equal mass concentrations of each particle fraction included were prepared and analysed (mixes 5–8). The exact mixing ratios of all samples are given in Tables S1 and S2 in the SI, presented as mass concentrations, particle number concentrations and percentages. Additional information on analytical performance, practical considerations, and sample requirements of the investigated techniques is summarised in Table S3 (SI), providing a concise overview to support method selection for specific analytical questions.

### 2.3 Dynamic light scattering (DLS)

Dynamic light scattering (DLS) was used to determine the hydrodynamic diameter (*z*-average) and size distributions of colloidal gold nanoparticles. The technique relies on the Brownian motion of particles, which causes fluctuations in the intensity of light scattered when the sample is illuminated with a laser. These intensity fluctuations are recorded over time as an autocorrelation function. From the initial exponential decay of this correlation function, the translational diffusion coefficient is obtained. Applying the Stokes–Einstein equation, the intensity-weighted harmonic mean particle diameter (‘*z*-average’; *z*_av_) is calculated.

Measurements were conducted in accordance with ISO 22412 ^70^ on a Zetasizer Nano ZS instrument, software version 8.01.4906 (Malvern Panalytical, Worcestershire, UK) equipped with a 633 nm He–Ne laser. Disposable micro-UV cuvettes (Brand, Germany) were used, and measurements were performed at a backscattering angle of 173°. Samples were equilibrated for 2 min at 25 °C prior to measurement. As the dispersant was a 2 mM sodium citrate buffer (pH 6.6), water-like properties were assumed, namely a refractive index of 1.330 and a viscosity of 0.8872 mPa s at 25 °C. For the gold nanoparticles, a complex refractive index of 0.20 + 3.32*i* at 633 nm ^71^ was applied in the calculations.

Instrument parameters such as measurement position, attenuator, and number were set to ‘auto’ mode, allowing the software to optimise conditions for statistical reliability. Measurements were performed at least in triplicate with a minimum acquisition time of 60 s per run. For the low-concentration AUXS sample, where scattering intensity is limited and measurement uncertainty is higher, 3 × 3 replicates were employed to improve precision. Correlation functions were analysed using the instrument's general-purpose algorithm (non-negative least squares fit, cumulants analysis).

No hydrodynamic diameter was provided by the manufacturer for the concentration standard AUXS50. For the three materials AUXU30, 50, and 100, hydrodynamic reference values were specified as *z*-averages (*z*_av_) determined by photon correlation spectroscopy; however, no measurement uncertainties were indicated. Accordingly, *z*-averages were reported for the DLS measurements in Section 3.1 (AUXS 50) and 3.2 (AUXU30, 50, 100). To ensure comparability of the measurement results across the four analytical techniques, the intensity-weighted DLS distributions were converted into number-weighted values, and the corresponding number mean diameters were reported (Section 3.3). For these calculations, the software assumes dilute, monodisperse, spherical particles with negligible interparticle interactions, and requires the optical parameters of the particle material. The conversion of intensity-weighted size distributions into number-weighted distributions was performed using the instrument software based on Mie scattering theory. As a result, the calculated number-weighted distributions represent model-derived estimates and should be interpreted with caution, particularly for polydisperse samples or systems containing aggregates. However, because all particles investigated in this study were smaller than 100 nm and thus predominantly within the Rayleigh or near-Rayleigh scattering regime, the influence of the assumed optical parameters on the intensity-to-number conversion is expected to be comparatively limited relative to larger particle systems.

DLS measurements were carried out under conditions recommended by the manufacturer. Instrument-selected attenuator settings, count rates, and the instrument's internal quality criteria were used to verify appropriate measurement conditions and to exclude excessive sample concentration and multiple scattering.

The results of the individual measurements are summarised in an accompanying data publication.^72^

### 2.4 Particle tracking analysis (PTA)

Particle tracking analysis (PTA) was employed to determine the particle size and particle number concentration of colloidal gold nanoparticles by tracking their Brownian motion in suspension. Based on the assumption of spherical particles undergoing free diffusion, the hydrodynamic diameter based on the Stokes–Einstein equation was calculated from the mean squared displacement of individually tracked particles. In contrast to DLS, PTA provides number-weighted size distributions by analysing the trajectories of single particles visualised *via* scattered light.^73,74^

Measurements were conducted using a NanoSight NS300 instrument (Malvern Panalytical, UK) equipped with a 488 nm laser diode and a high-sensitivity CMOS camera. Besides the AUXS nanoparticle standard, which was measured undiluted, all other samples were further diluted with 2 mM sodium citrate buffer to reach the optimal particle concentration range (typically 10^6^–10^9^ particles per mL)^75,76^ and injected using a syringe pump into a low-volume flow cell at a controlled flow rate of 4.55 µL per min for 60 s at 25 °C. The resulting particle concentrations were in the order of 10^8^ particles per mL, which lies within the concentration range specified in ISO 19430 ^75^ and is also recommended by the instrument manufacturer for optimal measurement conditions. The dispersing medium was assumed to have water-like properties due to the low sodium citrate concentration.

For each sample, five replicate measurements were performed and the results were averaged and expressed as mean. At least 775 valid particle tracks were recorded per sample and analysed using the instrument software with ‘Finite Track Length Adjustment’ (FTLA) mode. The mean hydrodynamic diameter (based on the number-weighted distribution), the modal value, and the particle number concentration (in particles per mL) were directly determined by the instrument software (version 3.4) for monomodal distributions. For multimodal samples the mean values within the selected peak boundaries had to be calculated from the raw data (particle counts in individual bins). The results of the individual measurements are summarised in an accompanying data publication.^77^

### 2.5 Single particle-inductively coupled plasma-mass spectrometry (spICP-MS)

For a typical ‘single particle’ analysis, a highly diluted suspension of metallic nanoparticles is introduced into the ICP-MS and becomes vaporized, atomized and ionized in the generated plasma. The basic assumption is that from each nanoparticle a single pack of ions will be generated, resulting in measurable pulse signals of less than about 0.5 ms at the detector. From the resulting time scan, the signal intensities can be converted into particle sizes based on the mass of element per particle event if additional information about the composition, density and shape of the particle is available. For the latter, a spherical shape is commonly assumed. The number concentration can be obtained from the frequency of the particle events detected.^78–80^

An iCAP Q (Thermo Scientific, Waltham, Massachusetts, United States) equipped with a PFA-ST MicroFlow nebulizer and a quartz cyclonic spray chamber cooled to 2 °C was used for spICP-MS measurements. The dwell time was set to 3 ms and the total acquisition time was 2 minutes per sample. The pump flow rate was measured daily before and after each test series and varied between 0.28–0.30 mL per min.

The transport efficiency (TE) describes the fraction of the nebulized suspension which reaches the plasma and its correct determination is fundamental for the measurement of particle size and number concentration. Various methods can be used for determining the transport efficiency and were applied in previous studies.^26,63,81,82^ However, being the most common and easiest methods, both the particle size method (TES) and the particle frequency method (TEF) were used for spICP-MS calibration: in particular, TEF und TES calibration was applied in step 1 (for comparing the size and particle number concentration). For steps 2–4 only TES calibration was applied.

All samples were diluted with ultrapure water and measured in triplicate containing three replicate runs. AUXS50 was measured in a concentration of 50 ppt. The measured concentration of the monomodal AUXU-NPs was 54 ppt for 30 nm, 52 ppt for 50 nm and 106 ppt for 100 nm, respectively. For the bi- and trimodal mixtures in step 3 and step 4, the exact mixing ratios are given in the SI section (Table S1). These mix samples were then further diluted (1 : 10 000) with ultrapure water before spICP-MS measurement. Using the Thermo Scientific™ Qtegra software (version 2.10.4345.136) and the ‘npQuant’ plugin, the mean diameter (in nm) and particle number concentration (in particles per mL) of the gold nanospheres in each sample were automatically obtained, based on the measurement of at least 250 particles (monomodal suspensions as well as multimodal mixtures based on equal number concentration) and 100 particles (multimodal mixtures based on equal mass concentration) of each size fraction in the mix. For better understanding of the analytical performance of spICP-MS and for visualising the individual particle fractions in a mixture in a resulting histogram, one trimodal AuNP sample of AUXU30/50/100 (mix 4) was exemplarily evaluated by the RIKILT single particle calculation spreadsheet.^83^ Further information on the individual measurements results can be found in the accompanying data publication.^84^

### 2.6 Scanning electron microscopy (SEM)

SEM analysis involves scanning the surface of a solid specimen point by point and line by line with a focused electron beam. Depending on the acceleration voltage of the primary electron beam and the sample material, various signals are generated simultaneously. These signals – primarily secondary electrons, backscattered electrons and element-specific X-rays – are collected by specific detectors to generate compositional information and high-resolution images of the sample surface.

SEM imaging was carried out with a ‘Quanta 250 FEG’ field emission gun scanning electron microscope (FEI, Brno, Czech Republic) at 30 kV. Sample preparation and image acquisition parameters were chosen according to ISO 19749.^85^ In detail, 20 µL of each AuNP sample was applied in triplicate to a Si wafer (5 × 7 mm chip, Plano GmbH, Germany) pre-treated with 1% Alcian blue solution (Sigma, Germany) for positive charging of the substrate surface. After 1 h incubation at room temperature in a closed Petri dish (to avoid evaporation), the sample substrate was dipped five times in ultrapure water for the removal of unbound particles and dried in a stream of nitrogen. Depending on the density of the nanoparticle distribution on the sample substrate, a set of 5–20 images was recorded for each monomodal sample at suitable magnification levels (20 000×, 40 000×, 80 000× or 120 000×), so that the area of the particles' 2D projections had at least a minimal size of 100 pixels and the total particles number was at least 500.^85,86^ For bi- and trimodal samples, the magnification level of 40 000× was suitable for reliable recognition of the smallest particle fraction (AUXU30).

Automatic image evaluation was carried out using the Zeiss Zen-Software (v. 3.9.5) in order to obtain size and concentration information for at least 500 AuNPs. This number was considered sufficient based on experience gained form previous participation in ILCs.^26,28^ For determining the particle size (minimal Feret diameter, *F*_min_), only individual particles were included in the size calculations and the particles were automatically sorted into the three size classes by applying size filters (30 nm AuNPs – dark-yellow: 20–40 nm; 50 nm AuNPs – turquoise: 40.1–60 nm; 100 nm AuNPs – dark-red: 60.1–120 nm). The particle counts were visually inspected with regard to false counted agglomerates and manually corrected if necessary. The raw data from image analysis were processed using Excel for determining the descriptive statistic parameters and are summarised in an accompanying data publication.^87^

Although no absolute particle number concentration can be obtained with SEM under the chosen study setting, the relative ratio can be obtained by determining the density of the counted particles per unit area in the analysed fields of views from the selected image stacks. For this, the ratio of each particle fraction in a mix sample was calculated by dividing the absolute particle number of each AuNP size class by the total number of all counted particles. This semi-quantitative approach provides a comparative measure across the different mix samples.

### 2.7 Statistical data analysis

The results are presented as mean ± standard deviation where applicable. The difference between the mean values of the measurement results (size and particle concentration) and the certified value provided by the manufacturer's CoA (see Table 1) were calculated according to an JRC application note.^88^ If there was a significant difference between certified and measured value, the results are indexed in the following tables. In lack of an available standard deviation in the CoA, this calculation is omitted for the particle number concentration or the hydrodynamic diameter and relating data is also marked.

## 3 Results and discussion

### 3.1 Step 1: characterisation of the nanoparticle number standard (AUXS50)

To evaluate the performance of the four different analytical methods for nanoparticle quantification, certified standard materials have to be tested for quality control. Although no certified reference material (CRM) is currently available for concentration determinations, several manufacturers now offer particle size standards (reference materials, RM) that also provide number concentrations as certified values, *e. g.* LGCQC5050 (LGC Limited, UK). A nanoparticle number standard, which has been commercially available from NanoComposix since autumn 2023, was used for this study to obtain relevant analysis results for all four techniques. It can be regarded as RM, but not as CRM, which provides a higher level of documented certainty and traceability.

The relevant certified values for AUXS50 are listed in Table 2. However, noteworthy is the fact, that no standard deviation is given for the particle number concentration.

**Table 2:** Measured size (nm) and particle concentration (particles per mL) of the nanoparticle number standard (AUXS50)

	Size (nm)	Particle number concentration (particles per mL)
Certified	51 ± 1.9 (by TEM)	1.1 × 10^8^ (by spICP-MS)
SEM	50.6 ± 5.9	n. d.^a^
spICP-MS (TES)	51.0 ± 0.2	7.8 ± 0.3 × 10^7^^c^
spICP-MS (TEF)	45.5 ± 0.1^b^	1.1 ± 0.1 × 10^8^^c^
DLS (*z*_av_)	59.1 ± 1.6^c^	n. d.^a^
PTA	57.2 ± 2.9^c^	1.1 ± 0.1 × 10^8^^c^

a n. d. = not detected.

b Significant difference between certified and measured value.

c Due to the lack of a diameter or given SD a statistical comparison to CoA could not be applied.

#### 3.1.1 Particle size

As shown in Table 2, the particle size was accurately determined by SEM and spICP-MS using TES calibration. Applying TEF calibration, the particle size was underestimated by 9%. As expected, the apparent particle sizes obtained by DLS and PTA are typically larger than the core particle diameters certified for TEM, as both techniques measure the hydrodynamic diameter, which includes the surrounding hydration (solvation) layer. Since the manufacturer provided particle size data only for the core diameter (determined by TEM), no statement can be made regarding the measurement accuracy of DLS and PTA; however, the repeatability of both methods was satisfactory.

With a standard deviation of ± 2.9 nm for the PTA measurement, the minimum reproducibility requirements stipulated in ISO 19430 are satisfied. Furthermore, the particle number concentration was determined with accuracy. DLS also reports a hydrodynamic diameter. In this case, the measurement had to be performed under suboptimal conditions due to the low particle concentration optimised for spICP-MS analysis. Nevertheless, the achieved repeatability was satisfactory when compared to the requirements specified in ISO 22412,^70^ which states that the reproducibility for monodisperse particles of approximately 50 nm should be below 5%. The observed variability of 2.7% is therefore well within the specified limit.

#### 3.1.2 Particle number concentration

With a deviation of less than 1%, very good agreement with the CoA value for the particle concentration was obtained when measured with PTA and spICP-MS calibrated with TEF. However, the spICP-MS results were significantly dependent on the different calibration methods (TES/TEF) and by applying TES calibration for spICP-MS, the particle count was underestimated by 29%. Even though the two approaches should theoretically be equivalent, several studies reveal the opposite.^59,89–92^ Possible influencing parameters are diverse and include among other things the accuracy of size and mass concentration of the AuNP standard (including the presence of a potential ionic fraction) used for TEF/TES calibration of the instrument, the type of surface treatments of the nanoparticles, the choice of diluent, the selection of fitting curves when determining transport efficiency, the storage conditions, the dilution during sample preparation as well as particle losses due to particle adsorption on pipette tips, sample tubes and in the sample introduction system.^26^

With awareness of these parameters, some of them can be minimised. However, since not all parameters can be controlled the selection of the method used for determination of the transport efficiency remains dependent on the purpose of the measurement. Therefore, better results are normally achieved using TEF for the determination of particle number concentration and TES for the determination of particle size, since the TES is not affected by particle loss.^26^

During the preliminary investigation with the particle number standard (AUXS50), this material turned out to be unsuitable for our further investigations because of application-related reasons. On the one hand, no standard deviation for the nanoparticle concentration is given, which excludes a validated comparison of the measured results with the CoA value. Also, no hydrodynamic diameter is specified, which would be necessary for comparison of particle size measurements with DLS or PTA. Although the ‘AUXS’ standard is commercially available in three size classes (30 nm, 50 nm and 100 nm), its low particle number concentration (1.1 × 10^8^ particles per mL) was unsuitable for preparing sufficient quantities of bi- and trimodal mix samples for this cross-method comparison study. To ensure appropriate comparability and to meet the requirements of all applied nanoanalytical techniques, the higher-concentrated monomodal gold nanoparticles of the ‘AUXU’ series were used for the following investigation (step 2–4).

### 3.2 Step 2: characterisation of the monomodal AuNP samples

In the next step, the three monomodal materials intended for mixing in steps 3 and 4 were individually characterised for particle size and number concentration using the four analytical techniques. The resulting values were compared with the specifications given in the certificates of analysis (CoA).

#### 3.2.1 Particle size

Table 3 presents the results for the particle sizes of the three different AUXU samples measured by all four methods in comparison to the corresponding CoA value. When the associated measurement uncertainties are considered, the results obtained by spICP-MS (based on TES calibration) and SEM are consistent with the manufacturer's specifications.^88^ This compliance with the specifications confirms the reliability and accuracy of the applied measurement methods for characterising the particle size of the samples.

**Table 3:** Overview of certified and measured hydrodynamic and core diameters of the monomodal AuNP samples

	AUXU30 size (nm)	AUXU50 size (nm)	AUXU100 size (nm)
Certified: mean core diameter (TEM)	28.2 ± 0.6	49.8 ± 1.8	102.2 ± 4.2
spICP-MS (TES calibration)	29.2 ± 0.3	49.3 ± 0.1	100.4 ± 0.2
SEM	28.7 ± 0.8	49.6 ± 1.8	98.2 ± 3.5
Certified: mean hydrodynamic diameter (*z*_av_)	36	59	107
DLS (*z*_av_)	34.8 ± 0.3^a^	58.1 ± 0.3^a^	107.9 ± 0.9^a^
PTA	38.9 ± 2.5^a^	55.9 ± 0.7^a^	108.2 ± 2.2^a^

a Due to the lack of given SD in the certificate a statistical comparison to CoA could not be applied.

For DLS and PTA, particle diameters were provided by the manufacturer without corresponding standard deviations, complicating direct comparison with the measured data. The DLS measurements obtained in this study were in 97–99% agreement with the manufacturer's values, while the hydrodynamic diameters measured by PTA agreed with the DLS manufacturer values to 92–99%. Measurement uncertainties ranged from 0.5 to 0.9% for DLS and from 1.3 to 6.4% for PTA, fulfilling the ISO criteria for reliable measurements. Consequently, both methods produced results that are in very good agreement with the manufacturer's specifications.

#### 3.2.2 Particle number concentration

In Table 4, the concentration measurements obtained by spICP-MS and PTA are presented as a comparative overview. With regard to the particle number concentration, DLS and SEM were not included in this assessment, as these methods cannot directly provide number-based particle concentrations.

**Table 4:** Particle number concentration (particles per mL) of the monomodal AuNP samples measured with PTA and spICP-MS

Particle number concentration (particles per mL)	AUXU30	AUXU50	AUXU100
Certified	2.4 × 10^11^	4.2 × 10^10^	4.9 × 10^9^
spICP-MS^a^	1.4 ± 0.1 × 10^11^	2.7 ± 0.1 × 10^10^	3.0 ± 0.2 × 10^9^
PTA^a^	1.6 ± 0.1 × 10^11^	3.0 ± 0.3 × 10^10^	7.5 ± 1.2 × 10^9^

a Due to the lack of given SD in the certificate a statistical comparison to CoA could not be applied.

Overall, it can be observed, that the measured data deviate noticeably from the nominal concentration, namely in the range of 28–53%. With the exception of the PTA value for the AUXU100 sample, all determined particle number concentrations are smaller than the reference value. Our observation that measuring the particle number concentration of (metallic) nanoparticles is associated with greater deviations than particle size measurements has also been shown in other studies using spICP-MS,^61,64,68,93^ reporting even much greater deviations of more than 200%.^68^ Due to sample preparation (storage, dilution, *etc.*), the calculated particle count usually overrates the actual measured values. Furthermore, absorption phenomena on vessel walls and pipettes make it difficult to produce samples with exact particle concentrations. The up to one-million-fold dilution required for spICP-MS analysis makes pipetting errors more relevant than for the other methods due to the various dilution steps. Additionally, the used TES calibration influences the spICP-MS measurement and leads to lower concentration values than expected, as previously shown in step 1. Nevertheless, the fact that the results are in the same order of magnitude as the reference values (calculated from the mass concentration and particle sizes rather than determined experimentally) can be considered in good accordance with manufacturer's specifications.

### 3.3 Step 3 and step 4: characterisation of bi- and trimodal AuNP samples

In the third and fourth step of the study, bimodal and trimodal particle mixtures with equal particle number concentrations and equal mass concentration of particles were analysed. The purpose was to investigate the ability of each analysis technique to distinguish the particle fractions within the mixtures, and to obtain information about their size and number concentration.

#### 3.3.1 Particle size

Tables 5 and 6 show the sizes obtained by the four methods, expressed as mean diameter ± standard deviation. Here, an overall mean diameter (‘overall particle size’) could be determined with all four methods, which, however, has little significance regarding the composition of the mixture and the suitability of the method applied in order to characterise a nanoparticle mixture.

**Table 5:** Size (nm) results for all measured bi- and trimodal samples: mix 1–4 represent the results for step 3 (equal particle number concentration), mix 5–8 represent the results for step 4 (equal mass concentration). n. d. = not detectable

		DLS	PTA	spICP-MS	SEM
Mix 1 (30/50)	Overall particle size	41.6 ± 0.7	56.4 ± 1.4	39.3 ± 0.1	39.0 ± 12.8
	30 nm fraction	n. d.	33.6 ± 1.0	28.7 ± 0.1	28.2 ± 1.1
	50 nm fraction	n. d.	56.1 ± 0.7	48.9 ± 0.1	48.8 ± 4.8
Mix 2 (30/100)	Overall particle size	87.0 ± 6.1	108.6 ± 1.0	60.1 ± 0.9	68.3 ± 36.9
	30 nm fraction	n. d.	n. d.	28.5 ± 0.2	28.8 ± 3.1
	100 nm fraction	n. d.	98.0 ± 6.2	98.1 ± 0.4	99.3 ± 4.1
Mix 3 (50/100)	Overall particle size	88.6 ± 0.7	97.5 ± 1.2	64.2 ± 0.9	74.4 ± 26.2
	50 nm fraction	n. d.	56.0 ± 3.7	48.5 ± 0.1	45.5 ± 2.7
	100 nm fraction	n. d.	99.0 ± 2.8	97.6 ± 0.4	96.6 ± 4.7
Mix 4 (30/50/100)	Overall particle size	88.2 ± 0.7	93.7 ± 1.1	54.2 ± 1.0	59.1 ± 31.8
	30 nm fraction	n. d.	n. d.	28.5 ± 0.1	30.5 ± 2.4
	50 nm fraction	n. d.	55.8 ± 1.3	48.5 ± 0.2	46.0 ± 3.0
	100 nm fraction	n. d.	100.2 ± 3.8	97.6 ± 0.4	96.9 ± 3.8
Mix 5 (30/50)	Overall particle size	35.8 ± 1.3	47.3 ± 0.6	33.5 ± 0.2	33.8 ± 7.3
	30 nm fraction	n. d.	32.0 ± 0.7	28.9 ± 0.1	28.1 ± 2.5
	50 nm fraction	n. d.	56.0 ± 1.5	48.9 ± 0.1	49.0 ± 1.9
Mix 6 (30/100)	Overall particle size	74.8 ± 4.2	51.7 ± 1.8	31.4 ± 0.1	33.1 ± 15.1
	30 nm fraction	n. d.	34.6 ± 0.9	28.5 ± 0.7	29.9 ± 2.6
	100 nm fraction	n. d.	92.7 ± 6.7	98.5 ± 0.3	100.8 ± 3.6
Mix 7 (50/100)	Overall particle size	75.6 ± 1.1	70.7 ± 0.3	50.4 ± 0.3	52.8 ± 17.2
	50 nm fraction	n. d.	51.8 ± 1.2	48.7 ± 0.1	45.9 ± 2.9
	100 nm fraction	n. d.	93.5 ± 3.2	98.8 ± 0.4	93.9 ± 4.5
Mix 8 (30/50/100)	Overall particle size	64.1 ± 4.8	54.6 ± 2.4	34.3 ± 0.2	35.8 ± 11.6
	30 nm fraction	n. d.	31.8 ± 1.1	28.8 ± 0.1	32.4 ± 2.5
	50 nm fraction	n. d.	54.0 ± 1.2	48.7 ± 0.1	47.7 ± 3.0
	100 nm fraction	n. d.	90.7 ± 3.5	98.6 ± 0.6	95.6 ± 2.8

Particle number concentration (particles per mL) of the different mixes measured with PTA and spICP-MS

**Table:** 

		Mix 1 AUXU30/50	Mix 2 AUXU30/100	Mix 3 AUXU50/100	Mix 4 AUXU30/50/100
Step 3 (equal PNC)	Calculated	1.4 × 10^9^	1.9 × 10^8^	1.9 × 10^8^	2.7 × 10^8^
	PTA	1.0 ± 0.1 × 10^9^	1.7 ± 0.1 × 10^8^	1.8 ± 0.2 × 10^8^	1.7 ± 0.2 × 10^8^
	spICP-MS	1.50 ± 0.02 × 10^9^	2.1 ± 0.1 × 10^8^	1.9 ± 0.1 × 10^8^	2.9 × ± 0.1 × 10^8^

**Table:** 

		Mix 5 AUXU30/50	Mix 6 AUXU30/100	Mix 7 AUXU50/100	Mix 8 AUXU30/50/100
Step 4 (equal mass concentration)	Calculated	2.9 × 10^9^	2.5 × 10^9^	4.8 × 10^8^	1.9 × 10^9^
	PTA	1.6 ± 0.1 × 10^8^	8.5 ± 0.8 × 10^8^	2.9 ± 0.2 × 10^8^	6.9 ± 0.4 × 10^8^
	spICP-MS	1.8 ± 0.06 × 10^9^	1.3 ± 0.5 × 10^9^	2.8 ± 0.9 × 10^8^	1.21 ± 0.04 × 10^9^

The DLS results for the mixtures and their respective individual fractions are presented in the third column of Table 5. In both experiments – using equal particle number concentrations and equal mass concentrations – DLS was unable to resolve the sizes of the individual fractions. Instead, only an average overall particle size was obtained, which is inherent to the physical principle of the method. In dynamic light scattering, the scattering intensity of a particle scales with the sixth power of its diameter (*I* ∝ *d*^6^) in the Rayleigh regime.^74^ Even a small number of large particles (2% or less in mix 4 and 8) can therefore dominate the overall intensity and bias the apparent size distribution towards larger particle diameters. As a result, DLS produces an intensity-weighted distribution that disproportionately represents larger particles relative to their actual number concentration.

**Fig. 1:**
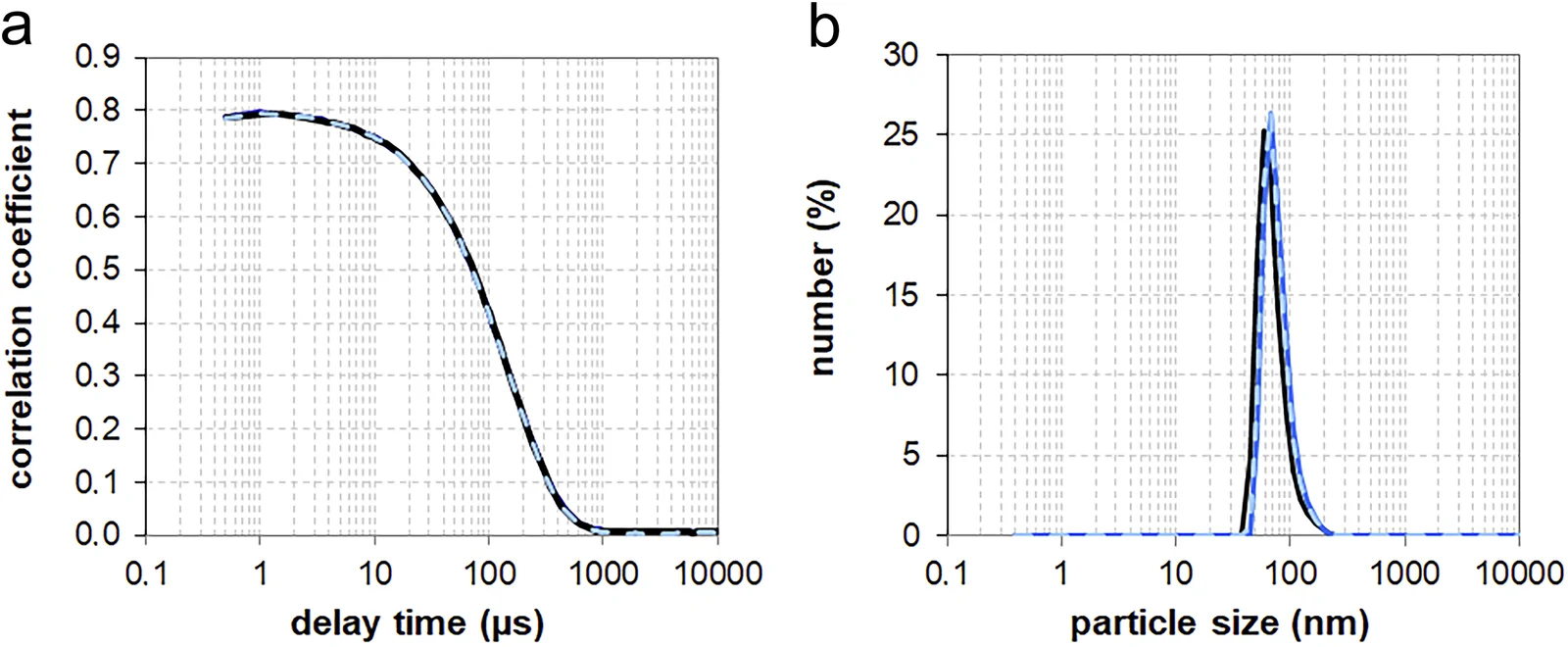
Representative DLS measurement (consisting of 3 single measurements) of the ‘equal mass’-based bimodal mixture (AUXU30/100, mix 6), (a) autocorrelation function, (b) particle number distribution obtained by Zetasizer software.

This is also reflected in the autocorrelation function (correlogram), which exhibited a smooth exponential decay characteristic of a monomodal distribution, without any indication of a second particle population. This demonstrates that fluctuations in scattering intensity caused by the smaller particles are effectively masked by the larger ones, resulting in a size distribution that essentially represents the average hydrodynamic size, biased towards larger particle sizes (Fig. 1).

In principle, DLS can identify individual populations within bimodal particle size distributions, provided that the size difference between the two populations is sufficiently large. The detectability depends on factors such as the size ratio of the two peaks, the particle number concentration, analytical model and the quality of the measurement.^55,71^ Studies of bimodal latex particle suspensions have shown that DLS can resolve distributions with particle size ratios of 20/100 nm, 30/100 nm, and 40/100 nm.^71^ The ability to detect the two populations also depends on their relative mass or particle number concentration. Using a single-angle setup, Malvern^73^ reported a minimal resolvable size ratio of approximately 3 : 1, whereas Zhu^94^ achieved improved resolution (∼1.5 : 1) by applying an optimised fitting algorithm for the autocorrelation function, which reduces the influence of noise at longer correlation times. This algorithm is not available in our current analysis, and therefore such enhanced resolution cannot be achieved in our measurements.

**Fig. 2:**
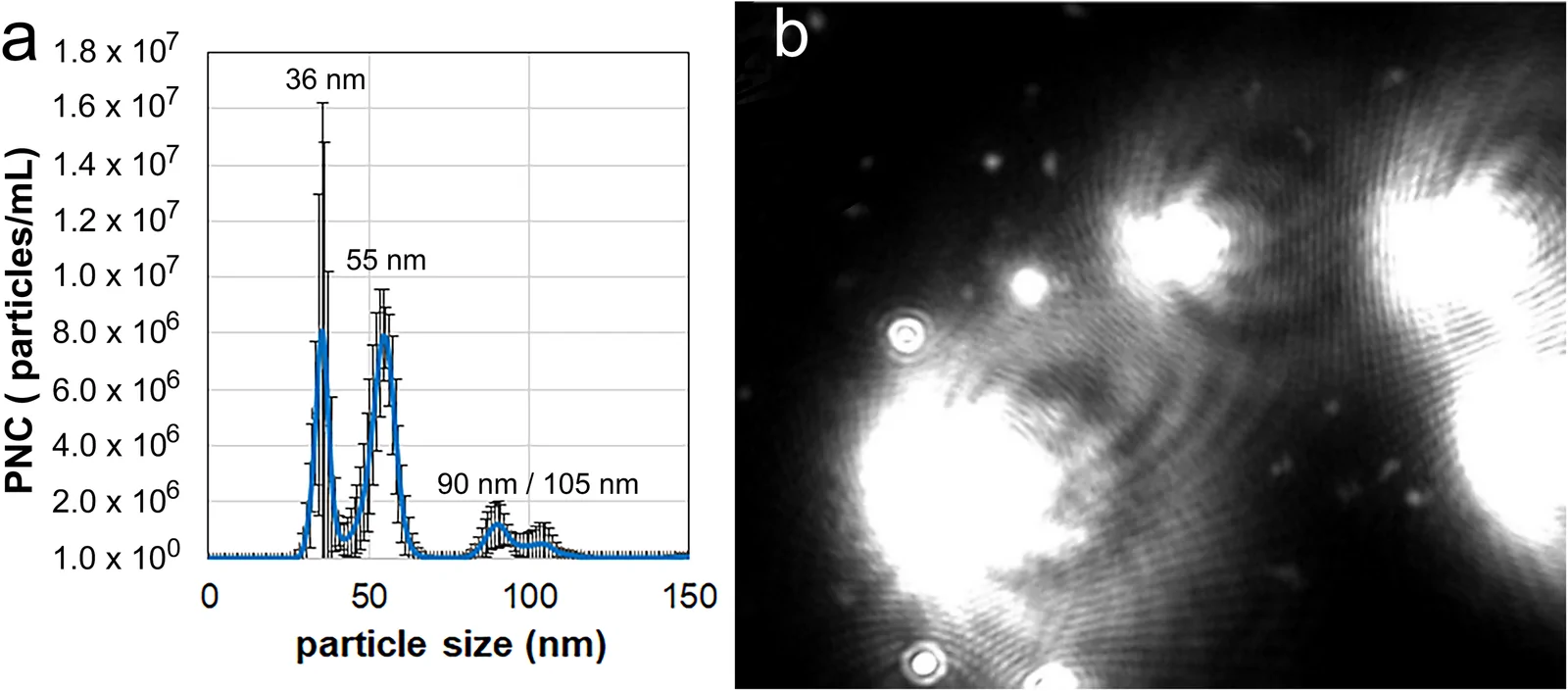
(a) Representative PTA measurement of the ‘equal mass’-based trimodal mixture (AUXU30/50/100, mix 8) (b) PTA frame showing a bimodal, ‘equal mass’-based mixture of AUXU30/100 nm particles (mix 6).

The PTA results are summarised in the fourth column of Table 5. For the number-based bimodal mixtures ‘mix 1’ and ‘mix 3’, both particle size populations were successfully identified. In contrast, in the bimodal mix 2 and the trimodal mix 4, the 30 nm AuNP fraction could not be detected, which can be attributed to the low relative proportion of small particles and the fundamental physical principles of light scattering. The latter also contributes to an inaccurate representation of the relative proportions of the individual fractions. This aspect is discussed in greater detail in Section 3.3.3. In contrast, for the mass-based mixtures (mix 5–8), all particle populations could be identified (see *e.g.* trimodal mix 8 in Fig. 2a). However, in most of the mixtures, the signal of an additional larger fraction with the size of approximately 105 nm was observed, which most likely corresponds to particle agglomerates. Fig. 2b shows an example of how the intense scattered light from the larger particles obscures the smaller ones, hindering their proper detection.

**Fig. 3:**
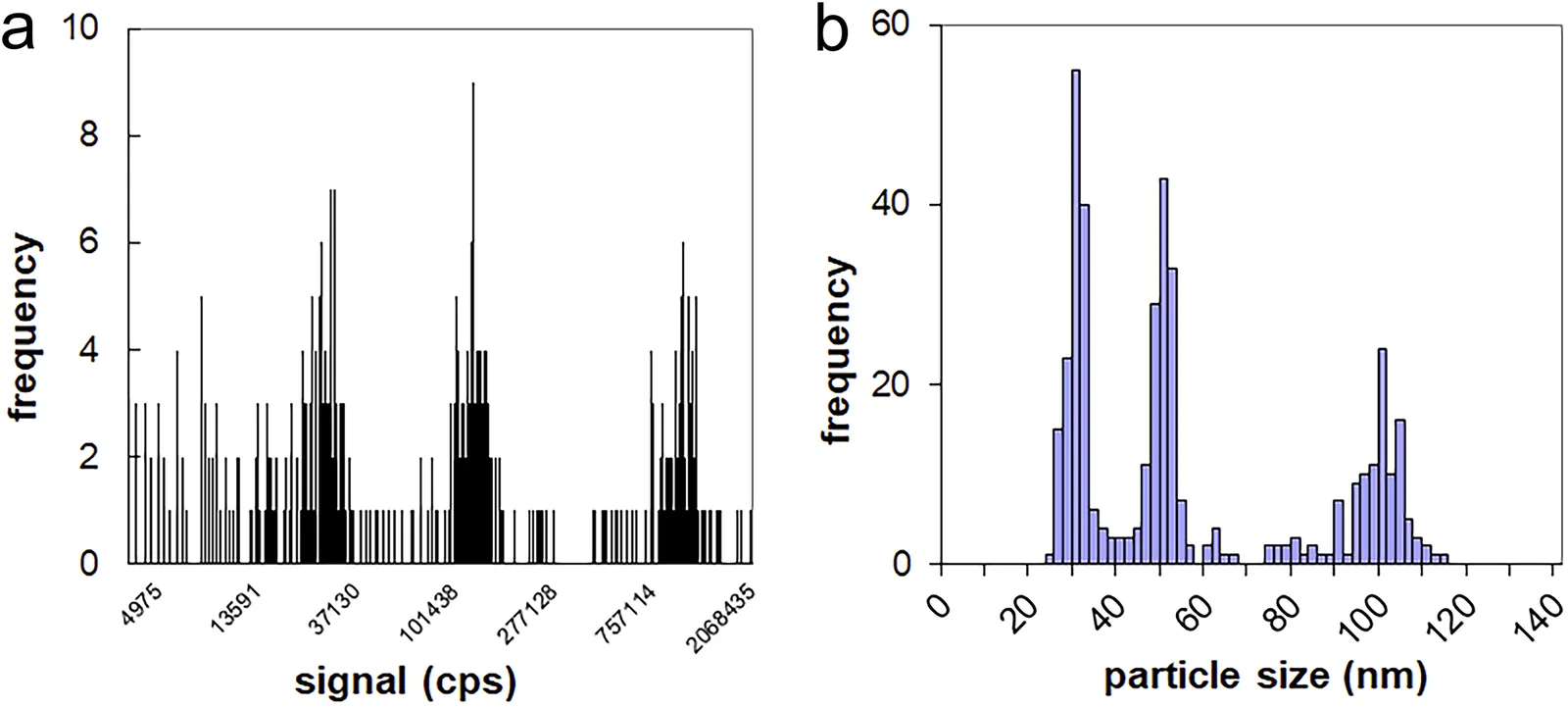
(a) Representative spICP-MS measurement signal of the ‘equal concentration’-based trimodal mixture (AUXU30/50/100, mix 4) and (b) the corresponding size distribution displayed as a histogram.

In contrast, the use of spICP-MS and SEM allowed the identification of each population within the various mixtures (column 5 and 6 of Table 5). The resulting sizes of each fraction were found to be in very good agreement with the results obtained in step 2 (monomodal AuNPs) and with the CoA's size specifications (according to ref. 88). In both step 3 and 4, spICP-MS and SEM yielded results with good reproducibility. The following Fig. 3 illustrates the spICP-MS signal detection and evaluation process by showing a typical signal frequency plot (Fig. 3a) and the corresponding size distribution histogram (Fig. 3b) for the measurement of AUXU30/50/100 (mix 4). In both, the individual size fractions are clearly distinguishable from one another. With the currently available evaluation algorithms, it is possible to distinguish nanoparticle populations with a size difference of 20 nm and more. However, Gao *et al.* showed that size discrimination limit for gold mixtures can be decreased to about 5 nm with resolvable component content of *<*10% using the kernel density estimation (FMKDE) model.^95^

**Fig. 4:**
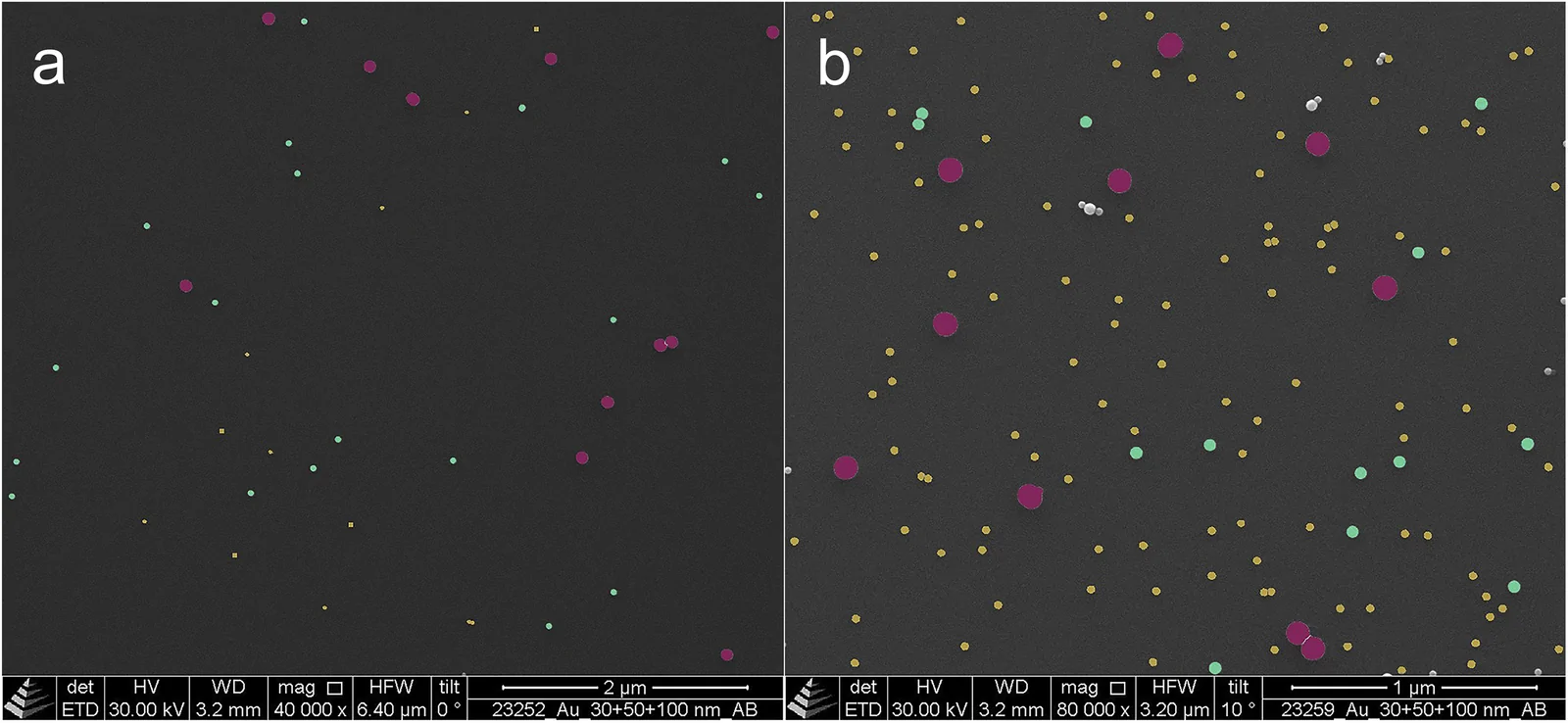
Representative SEM images of the trimodal mixture (AUXU30/50/100) with (a) equal particle number concentration (mix 4) and (b) with equal mass concentration (mix 8); the coloured particles represent the three different size fractions detected and measured by the imaging analysis software.

With SEM analysis the different sizes in the mixture were also easily identifiable, so that the resulting images could automatically be evaluated with the image processing software, as shown in Fig. 4.

#### 3.3.2 Total particle number concentration

As presented in Table 6, the total number concentration of the bimodal and trimodal gold nanoparticle suspensions could be determined very well using PTA and spICP-MS, similar to the monomodal gold nanoparticle samples of step 1. Here, the measured concentrations are in the same order of magnitude as the calculated nominal values. However, for some of the mixtures, a noticeable deviation from the calculated target value can be observed. As already discussed in chapter 3.2, this may be attributed to pipetting inaccuracies during the preparation of the mixtures and to particle loss (surface adsorption phenomena on the surface of sample vessels). Since the uncertainty of the CoA value is not specified, it was not possible to assess whether the observed deviations are statistically significant.

#### 3.3.3 Relative particle number concentration

**Fig. 5:**
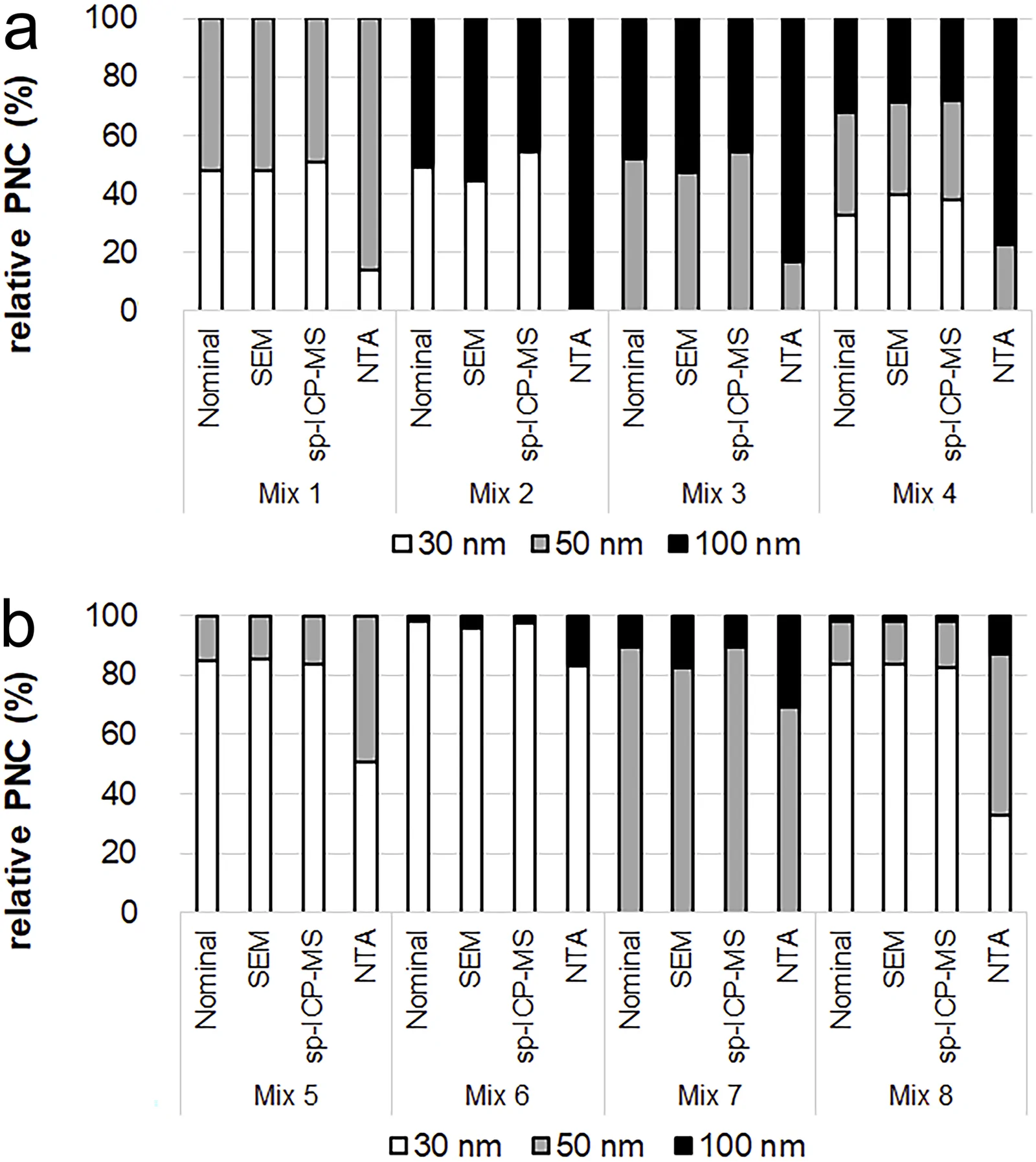
Comparison of the obtained relative particle number concentration (PNC) of the different size fractions in each mix sample: (a) mix 1–4 with equal particle number concentration (step 3) and (b) mix 5–8 with equal mass concentration (step 4). The ‘nominal’ columns represent the calculated target values.

To enable the inclusion of the semi-quantitative SEM results for this inter-method comparison, the particle number concentration results of PTA and spICP-MS analysis were also converted into relative ratios for each particle fraction in the different mix samples. The stacked bar diagrams in Fig. 5 show the obtained relative ratios in comparison to the calculated target concentration (‘nominal’) for all mix samples. The corresponding values, including the calculated deviation from the nominal value, are located in Table S4 (SI).

For all mix samples, the relative particle ratio calculated from the density of counted particles in the SEM images could be determined with only minor deviations in the range of 0.2–8 percentage points. This can be considered as a good result, given the difficulties in producing samples with exact particle number concentrations due to the factors already mentioned above. This is in accordance with an earlier ILC about the measurement of the relative number concentration of bimodal silica nanoparticle samples.^28^ Back then, our SEM lab (as one of 15 EM lab participants) obtained under similar preparations and analysis conditions also acceptable results with slightly larger deviations in the range of 4.5–12.7 percentage points.

Even though with SEM analysis reasonable information can be obtained about the relative PNC of nanoparticles, it is not possible to generalise that this approach works well for all particle materials and more complex samples. The crucial step lies in sample preparation when it comes to binding the nanoparticles homogeneously to the substrate surface (Si wafer). If this process is hampered, so that particles bind as aggregates very inhomogeneously or not at all, SEM analysis would lead to biased results, especially if an automated analysis is carried out.

The same applies for spICP-MS, which was generally suitable to depict the relative ratio of particle concentration. For particle number concentration-based mixtures (mix 1–4) the highest measured deviation from the target particle concentration ratio was about 5 percentage points difference of the 30 nm and 100 nm population in the bimodal mixture AUX30/50 (mix 2) as well as of the 30 nm fraction in the trimodal mixture AUX30/50/100 (mix 4). Mass-based measurements allowed for even more precise results of the population ratio in the mixtures (mix 5–8). Here, the highest deviation of the target particle concentration was found to be no more than 1.3%.

Although PTA is well suited for total particle analysis (see Section 3.3.2), the relative proportions of the particle populations were not accurately represented in any of the mixtures (Fig. 5). These observations result from the physical principles of light scattering and optical imaging (as already discussed in Section 3.3.1), which facilitate an easier and more reliable detection and tracking of nanoparticles. In contrast, smaller particles may be underrepresented if their scattering intensity falls close to the background noise level of the camera system, particularly in polydisperse samples (Fig. 5). This can introduce a systematic detection bias towards larger particles, especially when the samples show a wide size distribution or a large size difference between the particle fractions contained. Consequently, the quantitative accuracy of the number-based size distributions obtained by PTA analysis is affected by the detection sensitivity of the used instrument. In addition, the size resolution of PTA is inherently limited by the statistical uncertainty associated with Brownian motion analysis. In practice, particle populations generally need to differ by approximately 40–50% in diameter to be reliably distinguished. As a result, closely spaced particle populations may not be resolved into distinct peaks and can instead appear as a single broadened size distribution, depending on particle concentration, refractive index, and measurement conditions.^73,96^

As previously discussed, DLS cannot distinguish between the individual particle fractions within these mixtures and therefore cannot be used to determine their respective relative particle number concentrations. Even in mixtures where the number of large particles relating to the small ones is strongly reduced (equal mass mixtures) only an overall single measurement signal is observed.

## 4 Conclusions

In this study well-defined commercially available colloidal gold nanoparticle suspensions were used. However, due to the low initial particle number concentration and missing reference data in the CoA, these uniform standard suspensions posed a challenge with regard to the experimental design. Nevertheless, the study's systematic approach enabled a comparison of the results from four different nanoanalytical techniques used for the characterisation of nanoparticulate multimodal samples. It highlights the strengths and limitations of DLS, PTA, spICP-MS and SEM with regard to nanoparticle size and number concentration determination.

In nanoparticle characterisation, no single analytical technique can be considered universally applicable. Even when analysing nominally ‘pure’ nanoparticle systems, the choice of method must be guided by the specific scientific question, as each technique provides distinct and complementary information while exhibiting inherent limitations. This challenge becomes even more difficult and demanding when real-world or complex samples are analysed, where particle–particle interactions, agglomeration states, and matrix effects can significantly influence measurement results and potentially lead to discrepancies between methods.

For the chosen bi- and trimodal nanoparticle systems, spICP-MS and SEM are well suited for determining the mean particle size-based core diameter of each size fraction, whereas DLS and PTA provide information on the mean hydrodynamic diameter of the total particle population. While these parameters are not directly comparable, they represent physically distinct but complementary characteristics of the particles.

The total particle number concentration can be quantified using PTA and spICP-MS, as both techniques enable detection and counting of individual particles in suspension. In bimodal or trimodal mixtures, the individual particle size fractions can be resolved by spICP-MS, SEM, and, in certain cases, by PTA, depending on the size ratio and relative concentration of the fractions. The relative proportion of each fraction can be determined reliably using SEM and spICP-MS. DLS, in contrast, provides only an average hydrodynamic diameter across all particles, and due to the intensity-weighted nature of the measurement, larger particles contribute disproportionately, shifting the mean toward larger sizes.

However, the assessment of the informative value of the methods examined here for determining nanoparticle size and concentration relies on the assumption of spherical particles. Since individual events can be observed with PTA and spICP-MS, these methods can also be used to determine the PNC of non-spherical particles. Nevertheless, determining the accurate size and consequently size distribution is significantly more difficult for non-spherical particles (such as elongated, rod-shaped ones) and is not possible without additional information. Only SEM retains its validity even with irregularly shaped particles and still represents the method of choice for determining the size of non-spherical particles. In addition, it is important to note that spICP-MS is an indirect method, in which particle size is inferred from the measured particle mass, making accurate calibration critical for reliable results. Furthermore, different techniques yield method-specific characteristic particle size parameters and employ distinct statistical averaging principles (*e.g.*, number-weighted *vs.* intensity-weighted means). Consequently, direct comparison between techniques is challenging, and any attempt to normalise data across methods requires assumptions that may introduce systematic inaccuracies.

Despite these limitations, a wide range of complementary analytical approaches is available to achieve a more comprehensive and multidimensional characterisation of nanoparticles. Particularly useful could be correlative or hyphenated strategies, which combine complementary techniques to capture multiple aspects of a sample simultaneously. Such integrated strategies enhance the overall understanding of nanoparticle size distribution, number concentration and morphology, even though they do not resolve the inherent differences in size metrics between methods.

Further studies are required to validate analytical methods under realistic food conditions. These studies must take into account the occurrence of food matrix effects (*e.g.* protein corona formation, agglomeration/aggregation), sample heterogeneity, the need to identify nanomaterials within the food matrix, and, if necessary, to isolate them from other food components for further analysis. However, food-related certified test materials are currently not available.

## Author contributions

Conceptualisation: BH, AM, AKM, VG, EW. Data curation: BH, AM, AKM, VG, EW. Formal analysis: BH, AM, AKM, VG, EW. Investigation: BH, AM, AKM, VG, EW. Project administration: AM, BH, AKM. Validation: BH, AM, AKM, VG, EW. Visualisation: BH, AM, AKM, VG, EW. Writing – original draft: BH, AM. Writing – review & editing: BH, AM, AKM, VG, EW.

## Conflicts of interest

There are no conflicts to declare.

## Abbreviations

AFM: Atomic force microscopy
AuNPs: Gold nanoparticles
BET: Brunauer–Emmett–Teller
CEN: Comité Européen de Normalisation (European Committee for Standardization)
CoA: Certificate of analysis
CRM: Certified reference material
DCS: Differential centrifugal sedimentation
DLS: Dynamic light scattering
EDX: Energy dispersive X-ray (spectroscopy)
EFSA: European Food Safety Authority
EMPIR: European Metrology Programme for Innovation and Research
ENM: Engineered nanomaterial
EU: European Union
FIC: Food information to consumers
IHAT: Iron hydroxide adipate tartrate
ILC: Interlaboratory comparison
ISO: International Organization for Standardization
JRC: Joint Research Centre
NIF-LAG: Nanomaterials in Food – Laboratory Group
PTA/NTA: Particle/nanoparticle tracking analysis
OECD: Organisation for Economic Co-operation and Development
PEG: Polyethylene glycol
PNC: Particle number concentration
ppb/ppm/ppt: Parts per billion/million/trillion
PTA: Particle tracking analysis
RM: Reference material
SAXS: Small-angle X-ray scattering
SD: Standard deviation
SEM/TEM: Scanning/transmission electron microscopy
spICP-MS: Single particle inductively coupled plasma mass spectrometry
TEF/TES: Transport efficiency (determined by frequency/size)
TS: Technical specification
UV/vis: Ultraviolet-visible (spectroscopy)
VAMAS: Versailles Project on Advanced Materials and Standards
*z*_av_
: *z*-Average diameter

## Supplementary Material

NA-008-D6NA00161K-s001

## Data Availability

Four tables supporting this article have been included as part of the supplementary information (SI). Supplementary information: Tables S1–S4. See DOI: https://doi.org/10.1039/d6na00161k. Additionally, the primary data generated for each analytical technique in this study have been published as separate open-access data publications in OpenAgrar and are available *via* the following DOIs: https://doi.org/10.25826/Data20260202-153839-0 (for spICP-MS), https://doi.org/10.25826/Data20260209-093658-0 (for SEM), https://doi.org/10.25826/Data20260209-100314-0 (for DLS) https://doi.org/10.25826/Data20260209-102003-0 (for NTA).
