# Supplementary material for: Determination of particle size and number concentrations in gold nanoparticle mixtures: an inter-method study using four analytical techniques (DLS, PTA, SEM and spICP-MS)
Source: Nanoscale Adv. 2026 Jul 31. Online ahead of print. doi: 10.1039/d6na00161k (PMC13426056; doi:10.1039/d6na00161k)
Supplement: NA-OLF-D6NA00161K-s001 [file NA-OLF-D6NA00161K-s001.pdf]

This journal is © The Royal Society of Chemistry 2026

Supplementary Information: Determination of particle size and number concentrations in gold nanoparticle mixtures: An inter-method study using four analytical techniques (DLS, PTA, SEM and spICP-MS)

Birgit Hetzer, Alexandra Müller, Ann-Katrin Meinhardt, Volker Gräf and Elke Walz

Max Rubner-Institut, Federal Research Institute of Nutrition and Food,
Department of Food Technology and Bioprocess Engineering, Haid-und-Neu-Str. 9, 76131 Karlsruhe (Germany), corresponding author: birgit.hetzer@mri.bund.de

Table S1 Mass concentration (ppm) and particle concentration (particles/mL) of each AuNP size fraction of the prepared stock mixture samples (1:50-dilution of original samples) for the experiments with multimodal AuNP mixtures used for DLS, PTA and spICP-MS analysis. Further dilution steps for PTA and spICP-MS measurements are described in the methods section.

Sample	Particle concentration (mass-based and particle number-based)	AUXU30	AUXU50	AUXU100	Total (100%)
Mix 1	Mass conc. (ppm)	0,15<br>14.5%	0,91<br>85.5%	-	1.06
	Particle conc. (particles/mL)	6,8E+08<br>48.2%	7,3E+08<br>51.8%	-	1.4E+09
Mix 2	Mass conc. (ppm)	0,02<br>1.9%	-	1,06<br>98.1%	1,08
	Particle conc. (particles/mL)	9,5E+07<br>49.3%	-	9,8E+07<br>50.7%	1,9E+08
Mix 3	Mass conc. (ppm)	-	0,12<br>11.1%	0,96<br>88.9%	1,08
	Particle conc. (particles/mL)	-	9,64E+07<br>52.1%	8,86E+07<br>47.9%	1.85E+08
Mix 4	Mass conc. (ppm)	0,02<br>1.9%	0,12<br>10.9%	0,94<br>87.3%	1.08
	Particle conc. (particels/mL)	8,9E+07<br>32.9%	9,4E+07<br>34.9%	8,7E+07<br>32.2 %	2.7E+08
Mix 5	Mass conc. (ppm)	0,55<br>50.9%	0,53<br>49.1%	-	1.08
	Particle conc. (particles/mL)	2,4·E+09<br>85.1%	4,3E+08<br>14.9%	-	2.9E+09
Mix 6	Mass conc. (ppm)	0,55<br>50.3%	-	0,54<br>49.7%	1.09
	Particle conc. (particles/mL)	2,4E+09<br>98.0%	-	5,0E+07<br>2.0%	2.5E+09
Mix 7	Mass conc. (ppm)	-	0,53<br>49.4%	0,54<br>50.6%	1.07
	Particle conc. (particles/mL)	-	4,3E+08<br>89.5%	5,0E+07<br>10.5%	4.8E+08
Mix 8	Mass conc. (ppm)	0,36<br>33.8%	0,35<br>32.8%	0,36<br>33.4%	1.08
	Particle conc. (particles/mL)	1,6E+09<br>83.6%	2,9E+08<br>14.7%	3,3E+07<br>1.7%	1.9E+09

Table S2 Mass concentration (ppm) and particle concentration (particles/mL) of each AuNP size fraction in samples for the experiments with multimodal AuNP mixtures measured by SEM.

Sample	Particle concentration (mass-based and particle number-based)	AUXU30	AUXU50	AUXU100	Total (100%)
Mix 1	Mass conc. (ppm)	0.54<br>14.3%	3.22<br>85.7%	-	3.76
	Particle conc. (particles/mL)	3.3E+10<br>48.0%	3.6E+10<br>52.0%	-	7.0E+10
Mix 2	Mass conc. (ppm)	0.05<br>2.0%	-	2.60<br>98.0%	2.65
	Particle conc. (particles/mL)	4.8E+09<br>50,0%	-	4.8E+09<br>50,0%	9,6E+09
Mix 3	Mass conc. (ppm)	-	0.32<br>11.0%	2.60<br>89.0%	2.92
	Particle conc. (particles/mL)	-	4.7E+09<br>52.0%	4.3E+09<br>48.0%	9.1E+09
Mix 4	Mass conc. (ppm)	0.05<br>1.8%	0.32<br>10.9%	2.60<br>87.3%	2.97
	Particle conc. (particles/mL)	4.3E+09<br>32.4%	4.6E+09<br>35.2%	4.3E+09<br>32.4%	1.3E+10
Mix 5	Mass conc. (ppm)	1.62<br>50.0%	1.56<br>49.1%	-	3.18
	Particle conc. (particles/mL)	1.2E+11<br>85.1%	2.1E+10<br>14.9%	-	1.4E+11
Mix 6	Mass conc. (ppm)	1.62<br>50.5%	-	1.59<br>49.5%	3.21
	Particle conc. (particles/mL)	1.2E+11<br>98.0%	-	2.5E+09<br>2.0%	1.2E+11
Mix 7	Mass conc. (ppm)	-	1.56<br>49.5%	1.59<br>50.5%	3.15
	Particle conc. (particles/mL)	-	2.1E+10<br>89.6%	2.5E+09<br>10.4%	2.3E+10
Mix 8	Mass conc. (ppm)	1.62<br>34.0%	1.56<br>32.7%	1.59<br>33.3%	4.77
	Particle conc. (particles/mL)	8.0E+10<br>83.7%	1.4E+10<br>14.6%	1.6E+09<br>1.7%	9.6E+10

Table S3: Qualitative and quantitative comparison of the four nanoanalytical techniques used for this study

	**DLS** **Malvern Mastersizer Nano ZS**	**PTA** **Malvern Panalytical Nanosight NS300**	**spICP-MS** **iCAP Q**	**SEM** **FEI Quanta 250**
**Fundamental Principle & Method Type**				
What is the measurement principle?	Fluctuations of scattered light from Brownian motion of nanoparticles in dispersion	Tracking Brownian motion of individual nanoparticles in dispersion based on their scattered light	Mass-spectrometric detection of ion clouds generated from individual atomized and ionized nanoparticles	Scanning the surface of particles with a focussed electron beam induces interactions with the sample's surface; SEM images are based on emitted secondary electrons
What type of measurement method?	Ensemble measurement	Single particle measurement	Single particle measurement	Single particle measurement
Is it a screening or confirmatory method?	Screening method	Screening method	Screening method	Confirmatory method
Is regular calibration required?	No (but regular performance verification using reference materials is recommended)	No (but regular performance verification using reference materials is recommended)	Yes	No
What ISO standards are available?	ISO 22412:2025	ISO 19430:2024	ISO/TS 19590:2024	ISO 19749:2021<br>ISO 13322-1
**Sample Preparation & Procedure**				
What kind of samples can be analysed?	Particles, droplets, (bubbles) in liquid dispersions	Particles, droplets, (bubbles) in liquid dispersions	Particles in liquid suspension	Powders or dried suspensions
What kind of material can be analysed?	Organic and inorganic	Organic and inorganic	Inorganic<br>(metals, metal-oxides)	Organic and inorganic

	DLS<br>Malvern Mastersizer Nano ZS	PTA<br>Malvern Panalytical Nanosight NS300	spICP-MS<br>iCAP Q	SEM<br>FEI Quanta 250
How much time is typically required for the analysis of one sample (including preparation and data analysis)?	< 15 minutes	< 15 minutes	2-3 hours	2-3 hours
**Measurement Parameters & Performance Range**				
At what particle concentration range does the method typically operate?	ppb	Sample dependant (typically ~10^7–10^9 particles/mL)	ppt	ppm
Which particle size parameter is determined?	Hydrodynamic diameter, intensity-weighted; ensemble average<br>no distinction between primary particles and aggregates	Hydrodynamic diameter of individual particles; number-based distribution<br>no distinction between primary particles and aggregates	Equivalent mass-based spherical diameter of particles<br>no distinction between primary particles and aggregates	Geometric size (incl. shape parameters) based on the 2D-projection of the particle/aggregate<br>shape parameters measurable<br>distinction between primary particles and aggregates
What type of concentration can be determined?	No concentration measurement possible	Number-based total particle concentration (quantitative)	Number concentration and mass concentration (quantitative)	Relative particle concentration<br>(semi-quantitative)<br>(depends on preparation)
What is the typical measurable size range?	~1 nm – 1 µm<br>(sample-dependent: refractivity, viscosity and density)	~30 nm – 1 µm<br>(sample-dependent: refractivity, scattering intensity for monomodal samples)	~30 nm – 1 µm<br>(sample-dependent)	~ 5 nm – 1 mm
Is it possible to determine the size of individual particle fractions in complex / multimodal samples?	Limited<br>(if ratio of small to large particles is sufficiently high to generate adequate scattering intensity)	Possible<br>(if size populations are well separated and concentration is suitable)	Possible	Possible

	DLS<br>Malvern Mastersizer Nano ZS	PTA<br>Malvern Panalytical Nanosight NS300	spICP-MS<br>iCAP Q	SEM<br>FEI Quanta 250
Is the identification of the elemental composition possible?	No	No	Yes<br>element-specific analysis;<br>differentiation of various elements possible	Yes<br>element-specific, qualitative analysis with EDX detector possible
**Data & Expertise**				
How complex is data analysis?	Low	Low	Medium, time-consuming	High, time-consuming
What level of technical expertise is needed?	Low for simple routine samples / high for complex samples	Low for simple routine samples / high for complex samples	Medium for sample measurement / high for data analysis	Medium for sample analysis<br>high for complex sample preparation and (manual) data analysis
**Practical Application & Routine Use**				
Is the method suitable for routine analysis or high-sample throughput?	Yes	Yes	Yes	No
What are the most critical key points for analysis?	Large or highly scattering particles may obscure detection of smaller particles; Use of accurate refractive index and viscosity parameters	Large or highly scattering particles may obscure detection of smaller particles; Use of accurate viscosity	Determination of Transport efficiency: TEF or TES; element-specific → only few types of inorganic particles can be analysed<br>single element detection	Sample preparation (e.g. drying artefacts)<br>appropriate choice of the image magnification level

Table S4 Overview of the ratio and the resulting deviation Δ of the measured PNC results from the nominal (calculated) target values for all mix samples. Nominal values for PTA/spICP-MS and SEM are listed separately, as SEM samples with higher PNC were prepared individually.

Sample			PTA		spICP-MS			SEM	
	Size fraction	Nominal ratio %	ratio %	Δ	ratio %	Δ	Nominal ratio %	ratio %	Δ
Mix 1	30 nm	48.2	13.8	- 34.4	50.9	2.7	48.0	47.8	- 0.2
	50 nm	51.8	86.2	34.4	49.1	- 2.7	52.0	51.8	0.2
Mix 2	30 nm	49.3	0.1	- 49.2	54.4	5.1	50.0	44.6	- 5.4
	100 nm	50.7	99.9	49.2	45.6	- 5.1	50.0	55.4	5.4
Mix 3	50 nm	52.1	17.1	- 35.0	54.6	2.5	52.0	47.1	- 4.9
	100 nm	47.9	82.9	35.0	45.4	- 2.5	48.0	52.9	4.9
Mix 4	30 nm	32.9	0.0	- 32.9	38.0	5.1	32.4	34.6	2.2
	50 nm	34.9	22.8	- 12.1	34.1	- 0.7	35.5	27.2	- 8.0
	100 nm	32.2	77.2	45.0	27.8	- 4.4	32.4	24.6	5.8
Mix 5	30 nm	85.1	50.7	- 34.4	83.8	- 1.3	85.1	85.6	0.5
	50 nm	14.9	49.3	34.4	16.2	1.3	14.9	14.4	- 0.5
Mix 6	30 nm	98.0	83.1	- 14.9	97.6	- 0.4	98.0	95.8	- 2.2
	100 nm	2.0	16.9	14.9	2.4	0.4	2.0	4.2	2.2
Mix 7	50 nm	89.5	69.4	- 20.1	89.3	- 0.2	89.6	82.4	- 7.2
	100 nm	10.5	30.6	20.1	10.7	0.2	10.4	17.6	7.2
Mix 8	30 nm	83.7	33.0	- 50.7	82.6	- 1.1	83.7	83.6	0.8
	50 nm	14.6	54.3	39.6	15.5	0.9	14.6	14.7	- 2.5
	100 nm	1.7	12.7	11.0	54.3	0.2	1.7	1.7	1.7
